# NMR and MD Analysis of the Bonding Interaction of Vancomycin with Muramyl Pentapeptide

**DOI:** 10.3390/ijms23031146

**Published:** 2022-01-20

**Authors:** Rafał Ślusarz, Barbara Dmochowska, Justyna Samaszko-Fiertek, Krzysztof Brzozowski, Janusz Madaj

**Affiliations:** Faculty of Chemistry, University of Gdańsk, Wita Stwosza 63, 80-308 Gdańsk, Poland; basia.dmochowska@ug.edu.pl (B.D.); j.samaszko-fiertek@ug.edu.pl (J.S.-F.); krzysztof.brzozowski@ug.edu.pl (K.B.); janusz.madaj@ug.edu.pl (J.M.)

**Keywords:** NMR, NOESY, HSQC, vancomycin, muramyl pentapeptide, MD, hydrogen bond, trajectory analysis

## Abstract

The article describes an NMR spectroscopy study of interactions between vancomycin and a muramyl pentapeptide in two complexes: vancomycin and a native muramyl pentapeptide ended with D-alanine (MPP-D-Ala), and vancomycin and a modified muramyl pentapeptide ended with D-serine (MPP-D-Ser). The measurements were made in a 9:1 mixture of H_2_O and D_2_O. The obtained results confirmed the presence of hydrogen bonds previously described in the literature. At the same time, thanks to the pentapeptide model used, we were able to prove the presence of two more hydrogen bonds formed by the side chain amino group of L-lysine and oxygen atoms from the vancomycin carboxyl and amide groups. This type of interaction has not been described before. The existence of these hydrogen bonds was confirmed by the ^1^H NMR and molecular modeling. The formation of these bonds incurs additional through-space interactions, visible in the NOESY spectrum, between the protons of the L-lysine amino group and a vancomycin-facing hydrogen atom in the benzylic position. The presence of such interactions was also confirmed by molecular dynamics trajectory analysis.

## 1. Introduction

**Figure 1:**
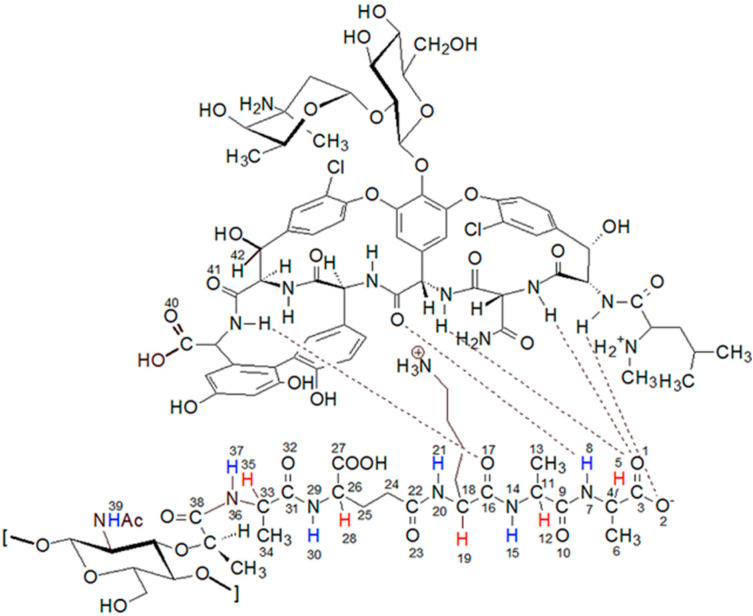
The structure of the complex formed by the peptidoglycan with the vancomycin binding pocket.

Vancomycin is one of the most important glycoprotein antibiotics and, thanks to its effectiveness, it is often called a last resort antibiotic. For over 60 years it has been used to treat infections with bacteria such as: *Staphylococci*, *Streptococci*, *Enterococci*, and *Pneumococci* as well as *Corynebacterium*, *Listeria*, *Bacillus* spp., *Clostridia*. It was first isolated by Dr. E.C. Kornfield, an organic chemist at Eli Lilly, from a fungus called *Streptomyces orientalis*. However, since then, many strains of bacteria resistant to vancomycin treatment have been isolated and described in the literature. In the mid-1980s, vancomycin-resistant *S. aureus* (VRSA) [1,2,3] and vancomycin-resistant *S. epidermidis* (VRSE) [4,5] strains appeared. The emergence of these vancomycin-resistant strains necessitates the search for new antibiotics that would be more effective in their treatment. The discovery of these vancomycin-resistant strains necessitates the search for new antibiotics that would be more effective in treating them. In the search for new, more effective antibiotics, the knowledge of their mechanism of action is extremely helpful. In the case of vancomycin, the most likely mechanism is the formation of a complex of vancomycin with the last two D-alanine residues in muramyl pentapeptide sequence [6,7]. This blocks the possibility of peptidoglycan cross-linking and, consequently, the expansion of the cell wall of gram-positive bacteria. The vancomycin-muramyl complex is stabilized by the hydrophobic van der Waals interactions and five hydrogen bonds formed between the L-lysine and subsequent two D-alanine residues, and the vancomycin binding pocket formed by the cyclic heptapeptide (Figure 1). To counteract this, bacteria have developed a defense mechanism involving the exchange of a terminal amino acid from D-alanine to D-serine or lactate. Especially in the latter case, this substitution causes significant decrease in effectiveness, which is explained by a weakening of the network of previously formed hydrogen bonds [8,9,10].

On the other hand, studies of the activity of vancomycin analogues modified in the sugar part show increased effectiveness, as pointed out by Min Ge and co-authors [11]. As they suggest, the mechanism of action of such antibiotics could be different from the interaction with the terminal peptidoglycan fragment. This possibility is also confirmed by our research on the activity of aglycone against some reference strains, which, despite the presence of full structure of the cyclic heptapeptide, shows a significantly reduced effectiveness [12]. An additional mechanism of vancomycin’s action has been described in the literature that involves the inhibition of ribonucleic acid synthesis by changing the permeability of the bacterial cell membrane [13]. This all shows that more research is still needed to further elucidate the effects of vancomycin as an antibiotic.

## 2. Results and Discussion

Research aimed at elucidating the mechanism of the interaction of vancomycin with peptidoglycan forming the gram-positive bacteria cell wall has been conducted for many years. Initially, attempts were made to determine these interactions based on X-ray analysis [14]. Soon after the structure of the vancomycin molecule was fully elucidated, the groundbreaking work of Williams’ team appeared, in which they used NMR spectroscopy to study the interaction of D-Ala-D-Ala dipeptide with vancomycin [15]. Further work that tried to explain this mechanism used increasingly complex research models with an increasingly longer peptidoglycan chain, such as the Whitesides study [16], in which they used a tripeptide including lysine. In our study on this mechanism, we decided to use the muramyl pentapeptide derivative, the synthesis, and characteristics of which we described in our earlier work [17].

### 2.1. NMR Study

We used an NMR spectroscopy to analyze the interactions. As an environment, we decided to use H_2_O and D_2_O mixture in a volume ratio of 9 to 1. Although most of the vancomycin NMR analyzes, including the first full assignment of signals [18,19,20] and probably the newest [21], were performed with DMSO, this system differs from what we observe in nature. Although the spectrum of vancomycin in water does not have such well separated signals, the use of high-resolution 700 MHz ^1^H NMR spectra, in combination with the solvent mixture used, turned out to have great advantages. The group of H-N amide signals and peptidoglycan Cα-H proton signals were completely separated, which made it possible to accurately track changes in their chemical shift on the spectrum and changes in the structure of the signals. We recorded the NMR spectra of two muramyl pentapeptides. The first is native pentapeptide with C-terminal D-alanine (MPP-D-Ala) and the second with D-serine (MPP-D-Ser) suggested in the literature [22]. For both pentapeptides, the spectra were also recorded after the addition of vancomycin in a molar ratio of 1:1. Moreover, for the native pentapeptide MPP-D-Ala, the spectrum was recorded after the addition of vancomycin in a molar ratio of 1:0.5. It allowed us to observe interesting effects confirming the influence of vancomycin concentration on the change of the structure of peptidoglycan signals.

**Figure 2:**
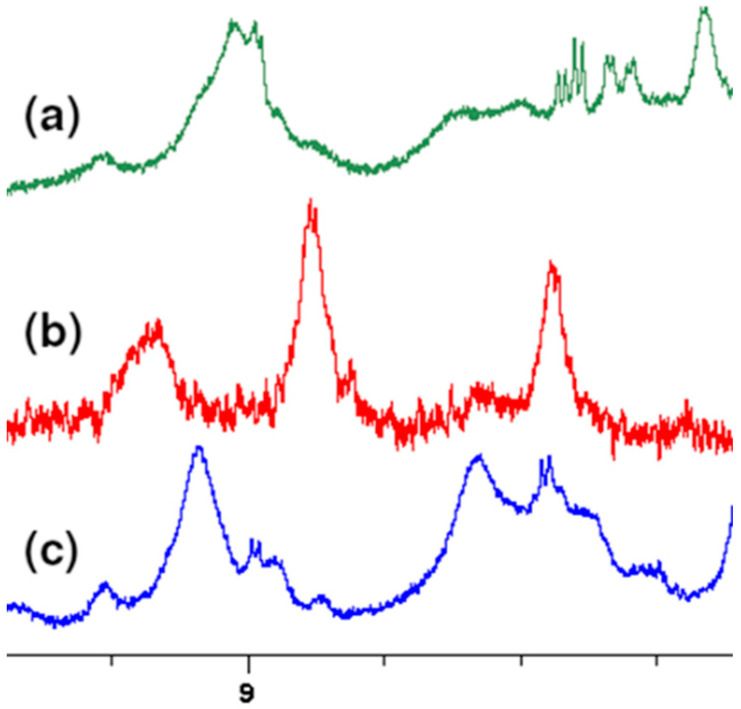
Fragment of overlapping ^1^H NMR spectra of: (**a**) MPP-D-Ser, (**b**) vancomycin, (**c**) MPP-D-Ala.

At the highest values of chemical shifts, signals from the 3 OH groups located on the aromatic ring of vancomycin are observed. Although these signals are broad, we can observe clear changes in their position as well. This is clearly seen in Figure 2, showing this fragment of the spectrum.

**Figure 3:**
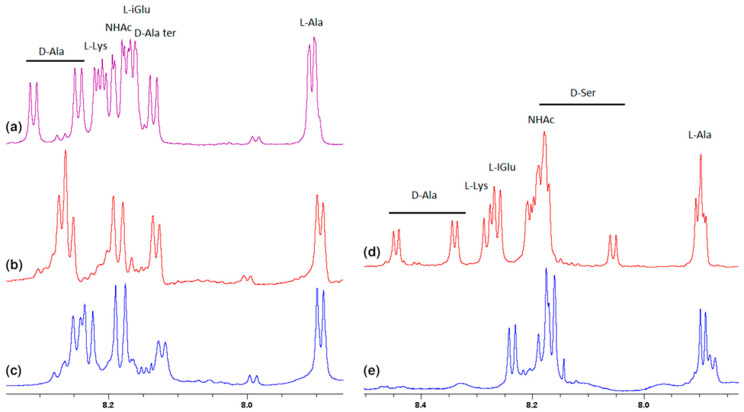
Fragments of the ^1^H NMR spectra of: (**a**) MPP-D-Ala, (**b**) mixture of MPP-D-Ala and vancomycin in a ratio of 1:0.5, (**c**) mixture of MPP-D-Ala and vancomycin in a ratio 1:1, (**d**) MPP-D-Ser, (**e**) mixture of MPP-D-Ser and vancomycin in a ratio of 1:1.

Much more characteristic changes can be observed in the region where the muramyl pentapeptide chain amide protons signals occur, which is shown in Figure 3.

Analysis of these spectra shows that both the L-Ala amide proton 37 (for atom numbering see Figure 1) and the muramic acid NHAc groups proton 39, undergo relatively slight changes in their positions. Considering the suggested mechanism of the interaction of vancomycin and muramyl pentapeptide, in which two D-alanine residues and oxygen atom from the L-lysine carbonyl group are involved, it is not surprising.

**Figure 4:**
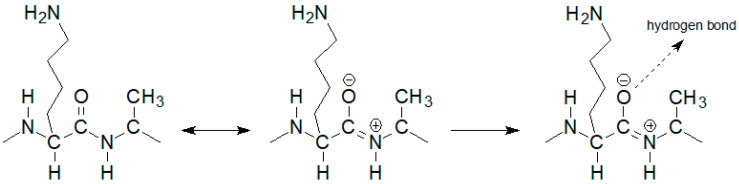
Diagram of hydrogen bond formation by the L-lysine amide group oxygen atom.

The notable change in the value of the chemical shift was observed for the amide proton 15 signal of the D-alanine amino acid residue penultimate in the pentapeptide chain. This is also seen in the MPP-D-Ser with vancomycin spectra. In the pure MPP-D-Ala spectrum, the signal of the amide proton of this D-alanine appears as the clearly separated two doublets (Δδ 0.065 ppm). When vancomycin was added, the two signals overlapped. It is even more spectacularly visible on the example of MMP-D-Ser. The presence of a hydroxyl group in D-serine causes the amide proton 15 signal to appear in the spectrum at higher δ values. When vancomycin is added, it clearly moves up the field. This behavior can be explained by Figure 4.

**Figure 5:**
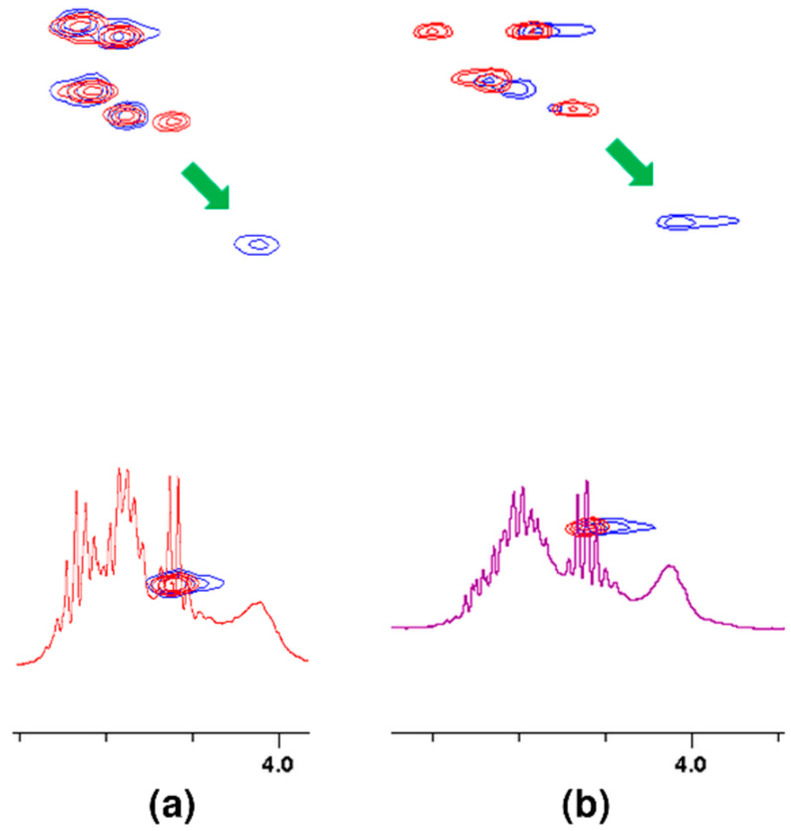
Fragments of HSQC spectra of 1:1 molar ratio of vancomycin mixtures with: (**a**) MPP-D-Ala, (**b**) MPP-D-Ser. Arrows show the shift of the signal after adding vancomycin.

If the oxygen atom 17 in the amide group begins to form a hydrogen bond, the electron density on it decreases. As a result, the order of the C-N partial double bond decreases, and the amide proton moves up the field. Such a situation is observed in the case of the carboxylic oxygen atom 17 of L-lysine and the amide hydrogen atom 15 of penultimate D-alanine, which leads to a shift of the signal of this proton observed in the spectrum. The analysis of the shift in the HSQC spectrum of the proton alpha 19 signal (Figure 5) confirms the participation of this oxygen atom in the formation of hydrogen bond. The acidity of the proton in the alpha position to the carbonyl carbon is well known in organic chemistry. The change in the electron density on this oxygen atom because of hydrogen bond formation reduces the acidity of this proton, which in the NMR spectrum results in its shift towards lower δ values. At the same time there is a shift of the carbon atom 18 towards higher δ values. We can observe it both in the spectra of the mixture of vancomycin with MPP-D-Ala and MPP-D-Ser, as shown in Figure 5.

**Figure 6:**
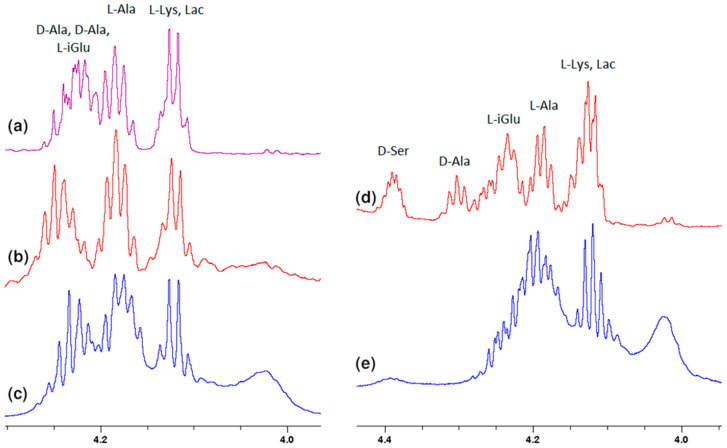
Fragments of the ^1^H NMR spectra of: (**a**) MPP-D-Ala, (**b**) mixture of MPP-D-Ala and vancomycin in a ratio of 1:0.5, (**c**) mixture of MPP-D-Ala and vancomycin in a ratio 1:1, (**d**) MPP-D-Ser, (**e**) mixture of MPP-D-Ser and vancomycin in a ratio of 1:1.

Analogously to oxygen atom 17 of amide group of L-lysine, terminal D-alanine, or D-serine carboxyl oxygen atoms 1 and 2 are involved in the hydrogen bond formation with vancomycin. As in the case of L-lysine, the spectra show analogous shifts of alpha proton 5. The presence of a hydroxyl group in D-serine makes this shift more spectacular in this case. Fragments of ^1^H NMR spectra illustrating this phenomenon are shown in Figure 6.

In addition to the two carboxyl oxygen atoms of the muramyl pentapeptide, the amide hydrogen 8 of the terminal D-alanine or D-serine is also involved in binding to vancomycin. According to the literature [23] for these atoms, this should result in a signal shift down the field. The analysis of the spectra in Figure 3 confirms the expected changes, but they have a smaller range and, similarly to the previous one, due to the specifics of the spectra, they are better visible for D-serine.

**Figure 7:**
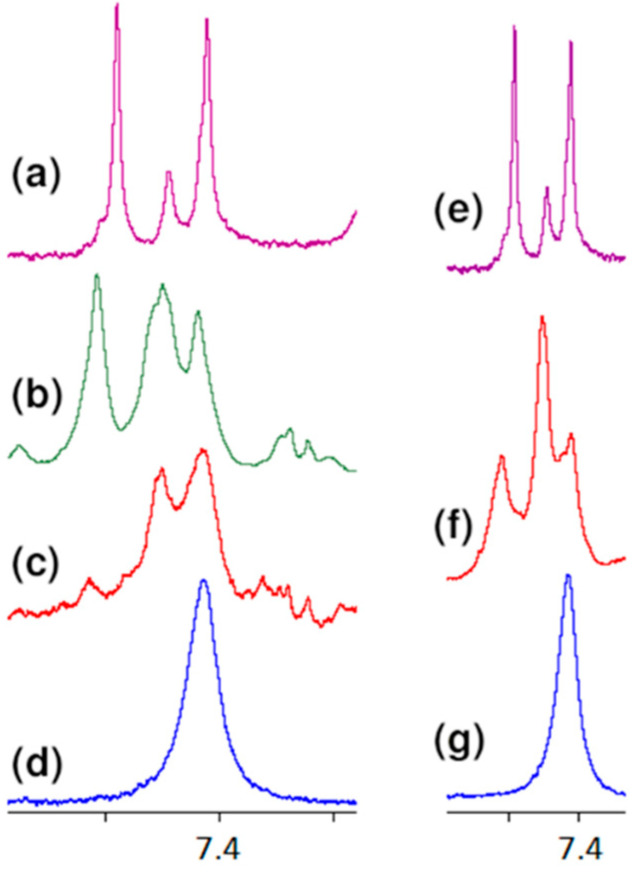
Fragments of the ^1^H NMR spectra of: (**a**) vancomycin, (**b**) MPP-D-Ala and vancomycin in molar ratio 1:1, (**c**) MPP-D-Ala and vancomycin in molar ratio 1:0.5, (**d**) MPP-D-Ala, (**e**) vancomycin, (**f**) MPP-D-Ser and vancomycin in molar ratio 1:1, (**g**) MPP-D-Ser.

All these observations presented above confirm the contribution of carboxy oxygen atoms and amide hydrogen in the formation of a complex with vancomycin presented in the literature. However, further analysis of the spectra indicates the formation of one more hydrogen bond, previously not described in the literature. Observing the position of the proton signal of the L-lysine amino group, it can be clearly seen that after adding vancomycin it moves down the field (Figure 7). The analysis of spectra with different vancomycin concentration (b and c) turns out to be useful in this case. It is clearly seen that the signal resulting from the interaction increases with increasing vancomycin concentration.

An analogous shift of the signal of protons of this group in the ^1^H NMR spectrum is observed in the case of MPP-D-Ser (Figure 7f).

**Figure 8:**
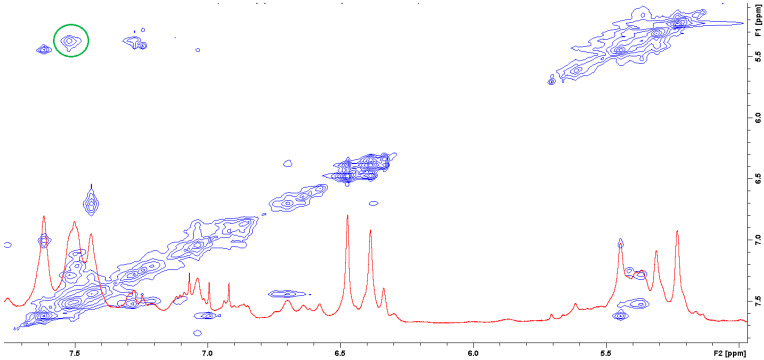
NOESY spectrum of the mixture of MPP-D-Ala and vancomycin in molar ratio 1:1 (the green circle marks the cross-peak indicating the through-space interaction of the protons of the amino group of L-lysine and the benzyl proton 42 of vancomycin).

**Figure 9:**
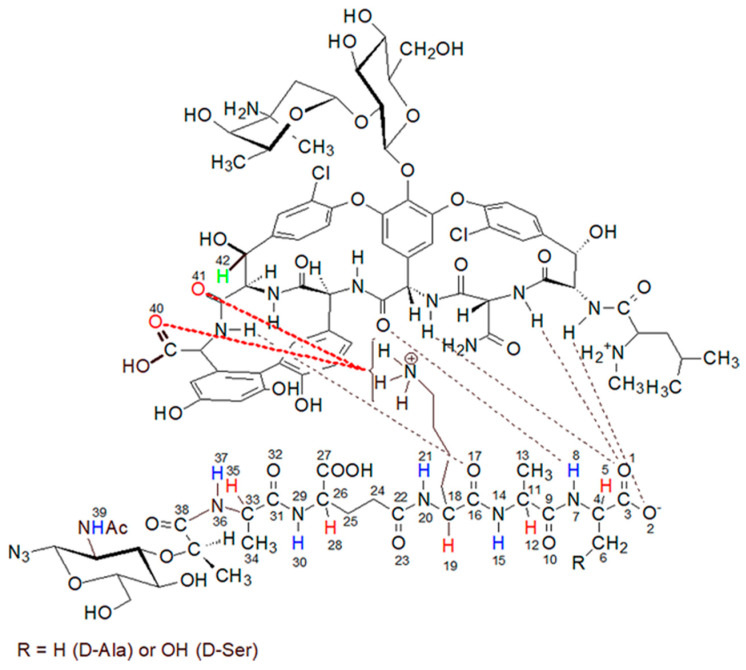
The proposed structure of the muramyl pentapeptide-vancomycin complex (MPP-D-Ala), considering the additional hydrogen bond (marked in red) between the amino group of lysine and the oxygen of the carboxyl and amide groups of vancomycin.

The question of which vancomycin group is hydrogen bonded by the L-lysine side chain amino group could be answered by analysis of the NOESY spectrum (Figure 8). It shows a cross-peak indicating the through-space interaction of the protons of the amino group of L-lysine and the benzyl proton 42 of vancomycin (in Figure 9 it is marked in green) resulting from the hydrogen bond formed between the amino group of L-lysine and the oxygen atoms 40 and 41 of the carboxyl and amide groups of vancomycin, as shown in Figure 9.

While reviewing the literature on the interaction of vancomycin with muramyl pentapeptide, we did not find information about this type of interaction. This may result from the previously used research models, in which this group was ignored or even protected with an acetyl group [24].

### 2.2. MD Study

The possibility of this binding did not come as a surprise to us. During the work on the material that we had previously published [25], we observed the formation of such a complex in molecular dynamics (MD) simulations. In MD, after initial geometry optimization, the distance between L-lysine side chain amine group and vancomycin C-terminal part was favorable for hydrogen bond formation.

**Figure 10:**
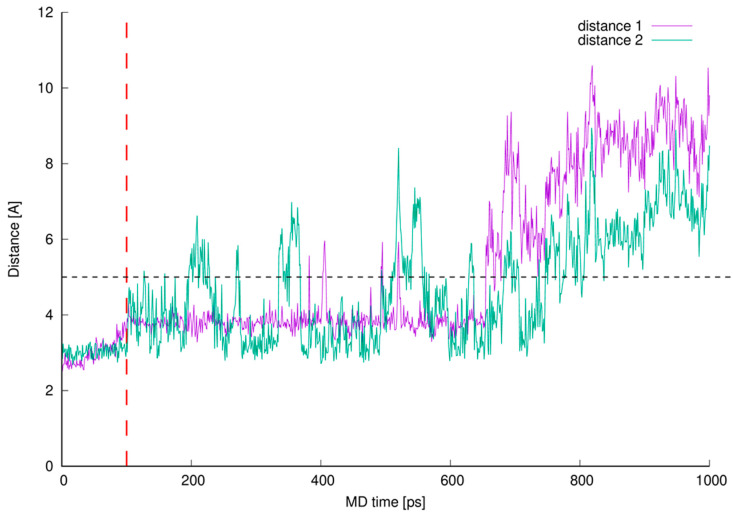
Newly described interactions’ measured lengths. The interacting parts are L-lysine side chain amine group and vancomycin oxygen atom 40 (**distance 1**) or L-lysine side chain amine group and vancomycin oxygen atom 41 (**distance 2**).

During the MD simulations we monitored existence of the expected hydrogen bonds between vancomycin and muramyl pentapeptide MPP-D-Ala (marked in Figure 1 and Figure 9) and, additionally, the distances between L-lysine side chain amine group and any of the potential proton acceptors: carboxyl oxygen atom from vancomycin C-terminus or carboxyl oxygen atom from its neighboring amide group (atoms 40 and 41, respectively; for atom numbering see Figure 9). From the MD run, a multiplots of those distances were prepared (Figure 10).

Lengths of newly described interactions were measured as a virtual distance between center of mass of L-lysine side chain amine group (involving four atoms) and center of mass of two oxygen atoms from the vancomycin C-terminal carboxyl group (distance 1) or previously described amine group and the oxygen atom 41 included in the amide group next to the vancomycin C-terminus (distance 2).

Initial 100 ps of the MD run (TDMD; see the Section 3) in Figure 10 is ended with red vertical, dashed line and is not considered as a part of actual MD therefore the distances between interacting parties in this region of the plots are not discussed here.

From the beginning of free, unrestrained MD, both distances between newly discovered centers of interactions are kept just below 4 Å, which is suitable for hydrogen bond formation. After 0.6 ns of MD these identified hydrogen bonds are disturbed and broken—the values of monitored distances are growing above 5 Å. Moreover, from the plots in Figure 10 one can see, that the distance 1 is even more often preserved on constant level than distance 2 which confirms higher adaptative conformational flexibility of vancomycin C-terminal carboxyl group then amide group involved in vancomycin macrocyclic peptide backbone.

**Figure 11:**
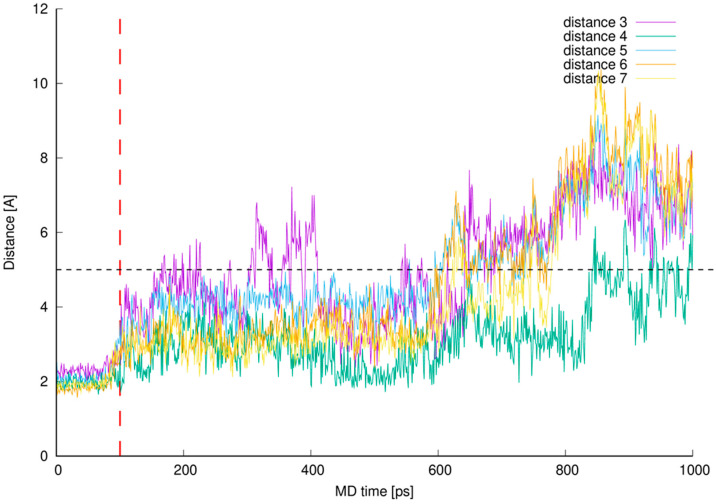
Control distances confirming hydrogen bonds presence in vancomycin-pentapeptide complex [14,15,26]. The interacting parts are hydrogen atom from the vancomycin C-terminal neighboring amide group and L-lysine backbone (amide) oxygen atom (**distance 3**), pentapeptide C-terminal D-alanine amide hydrogen and mid-vancomycin amide oxygen atom (**distance 4**), pentapeptide C-terminal D-alanine carboxyl group and three subsequent amide hydrogen atoms from the vancomycin macrocyclic peptide backbone (**distances 5, 6 and 7**)—all marked as a black dashed lines in Figure 1 and Figure 9.

The favorable distances between interacting parties are not preserved during whole MD which suggests that the interacting parties are observed only in some local energetic minimum induced by the initial TDMD. One can observe the presence of the hydrogen bonds discussed but the MD run in our investigation does not confirm their presence during the whole MD. It is caused by the full conformational freedom of all interacting parties and is expected. The confirmation of this can be found in Figure 11.

In Figure 11 one can see control distances measured between known and expected points of interactions between vancomycin and pentapeptide fragment complexed during vancomycin active bonding to the peptidoglycan cell wall [14,15,26] resulting in formation of five hydrogen bonds. If the line representing any distance is being plot below the contractual 5 Å limit, we assume (and can visually confirm) that the hydrogen bond is present in this moment of our MD simulation. Presence of these hydrogen bonds during MD simulation confirms proper model building and stable vancomycin-muramyl pentapeptide interaction.

Before 0.6 ns of MD simulation all monitored distances are kept at the expected level—below 5 Å—excluding distance 3. The distance 3 values are erratic and only for two thirds of the 0.6 ns of MD discussed is kept below 5 Å. This can be explained by the fact that this hydrogen bond is formed between hydrogen atom from the vancomycin C-terminal neighboring amide group and L-lysine amide group oxygen atom. The hydrogen atom discussed belongs to the amide group involved in hydrogen bond formation described as distance 2 in Figure 10. Wherever muramyl pentapeptide L-lysine residue is being pulled apart from vancomycin (from its C-terminus) both distances are increased—the hydrogen bonds represented by distances 2 and 3 are disrupted and possibly, temporarily terminated.

Where the control distances are increased above our contractual 5 Å threshold the hydrogen bonds are being broken. In the simulated complex this is caused by freeing the muramyl pentapeptide from the vancomycin binding cavity partially (between 0.6 and 0.8 ns of MD) or as a whole (after 0.8 ns of MD). We did not continue the MD after this point and did not impose any restraints to keep the ligand in the position meeting crystallographic criteria in active vancomycin-ligand complex.

From the analysis of the trajectory of complex simulated one more critical distance got our attention. This is the distance between L-lysine side chain amine group protons and aliphatic hydrogen atom 42 (HB). This specific hydrogen atom appeared to induce proton-proton interaction observed in NOESY spectrum (Figure 8).

**Figure 12:**
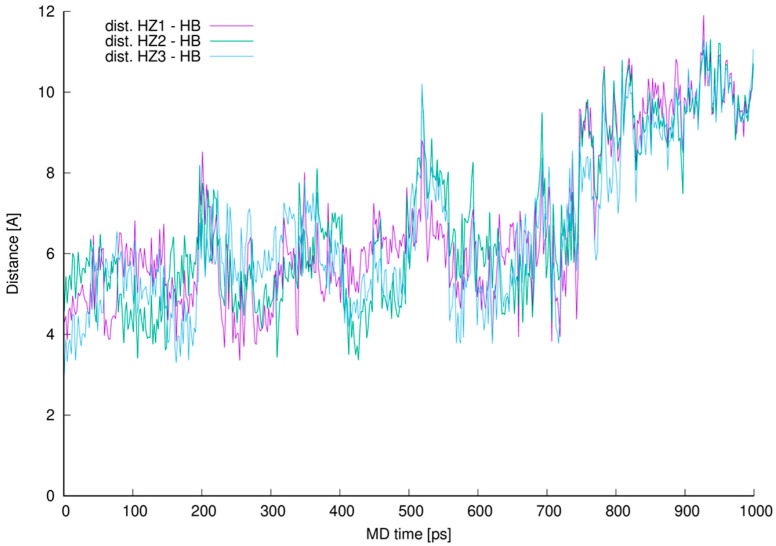
Distances between vancomycin hydrogen atom 42 (HB) and every L-lysine side chain amine group (HZ1, HZ2 or HZ3).

We prepared a plot illustrating how close those protons were during whole MD and presented it in Figure 12.

The distance analysis shows that at almost any point in the MD run at least one, most of the time two hydrogen atoms forming L-lysine amine group, is in the near vicinity to the aliphatic hydrogen atom 42 discussed. This makes it possible to influence the proton chemical shifts as observed in the NOESY spectra marked in green in Figure 8.

The interactions are possible, and we confirmed their existence in manual inspection of the MD trajectory snapshots excluding 190–230, 500–550 and above 690 ps MD regions. In those specific intervals the distances were greater, and the possible proton–proton interactions could be weaker or impossible due to too great distance. For most of the MD time discussed in this work (in the 0.1–0.6 ps interval of the MD it is almost 72% of the MD time) and even after the time when we no longer observe new, here described hydrogen bonds (after 0.6 ns of the MD time), the possible proton–proton interactions were present.

**Figure 13:**
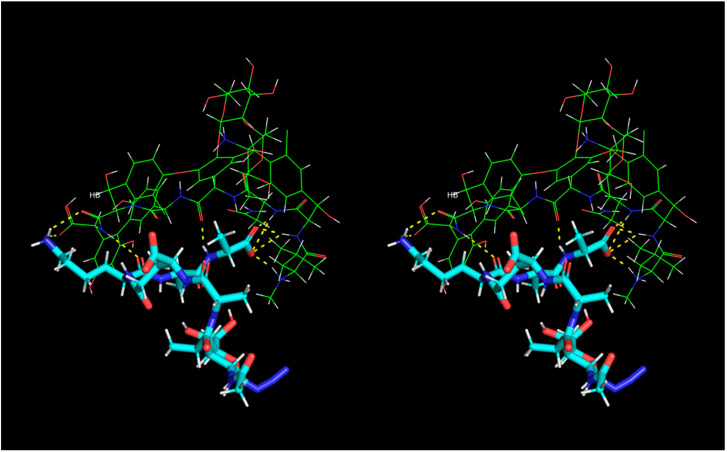
Stereo view (crossed eyes representation) of selected vancomycin-muramyl pentapeptide (MPP-D-Ala) MD snapshot with hydrogen bonds marked in yellow.

From the MD snapshots in which we could confirm presence of all discussed hydrogen bonds formed between muramyl pentapeptide and vancomycin we selected the complex with lowest total energy and presented it in Figure 13.

## 3. Materials and Methods

### 3.1. NMR Measurements

Amounts of vancomycin (12.1 mg, 8.1 μmol); vancomycin (6.1 mg, 4.1 μmol) and MPP-D-Ala (6.4 mg, 8.1 μmol); vancomycin (12.1 mg, 8.1 μmol) and MPP-D-Ala (6.4 mg, 8.1 μmol); vancomycin (13.1 mg, 8.9 μmol) and MPP-D-Ser (7.2 mg, 8.9 μmol) were dissolved in 0.5 mL of a mixture of H_2_O and D_2_O (vol. 9:1).

For the samples studied the following spectra were recorded: ^1^H and 2D TOCSY [27], NOESY [28,29] and ROESY [30] with over 4000 data points in f2 at mixing times of 80, 200 and 200 ms, respectively. Additionally for shift confirmation HSQC correlation spectrum was performed using standard sequence [31,32,33,34,35,36]. All measurements were carried out at Bruker 700 MHz spectrometer. All spectra were recorded at controlled temperature of 298 K using TXI inverse probe. To reflect natural conditions mixture of H_2_O and D_2_O at the ratio of 9:1 was used. Water signal suppression was achieved by the excitation sculpting protocol [37]. Obtained spectra were processed and analyzed with the usage of TopSpin 3.2 (Bruker BioSpin GmbH, Germany) software.

### 3.2. MD Simulations

For the MD simulations we have parameterized all non-standard groups and fragments for the AMBER [38] *ff03.r1* [39,40] and *glycam06g* [41,42] force fields as described in detail in [25]. In the crystallographic complex of vancomycin with di-acetyl-Lys-D-Ala-D-Ala ligand (PDB ID 1FVM, chain O) the ligand structure has been replaced with muramyl pentapeptide derivative described in [17,25] of the sequence: Mur-Ala-D-iGlu-Lys-D-Ala-D-Ala. Vancomycin vancosamine and the Lys side chain amine groups were protonated.

The pre-equilibrated water boxes, TIP3P [43] model, were added to the computational system. The rectangular water box containing interacting complex and water molecules size was 10 Å in each direction, which resulted the computational system containing about 1800 water molecules.

Geometry of the complex was optimized, and energy minimized. After initial geometry optimization, the hydrogen bond restraints defined to meet the crystallographic criteria were imposed to provide mutual adjustment of vancomycin and the muramyl pentapeptide in water solution.

Initial equilibration with adjustment was driven in the 300 K with the force constant 1 kcal/mol to achieve the crystallographic criteria in this Target Driven Molecular Dynamics (TDMD). In TDMD the interacting parties are guided towards a final (target) structure by means of steering forces in selected interacting points, which in this case were defined as expected hydrogen bonds. The initial TDMD was carried out for 100 ps and the force constant was applied for first 50 ps and then gradually reduced to 0 kcal/mol in the last 50 ps of TDMD.

Subsequently the simulation complex was submitted to the isothermal-isobaric (300 K) molecular dynamics (MD) in the AMBER suite of programs. For the MD we used SHAKE algorithm [44] with default time step 0.001 ps, Berendsen [45] bath coupling (1 ps) and 9.0 Å cut-off for non-bonded interactions, with PME [46,47,48].

Snapshots from the trajectory were taken every 1000 steps (1 ps) and analyzed over whole 1 ns of the run in the CPPTRAJ [49] module.

Total vancomycin-muramyl pentapeptide complex energy (AMBER’s EAMBER energy) was calculated by SANDER module of AMBER suite of programs.

## 4. Conclusions

It can be stated that the use of high-resolution NMR allowed to obtain spectra of vancomycin complexes with MPP-D-Ala and MPP-D-Ser in the mixture of solvents: H_2_O and D_2_O. We were able to confirm the presence of hydrogen bonds suggested in the literature. The analysis of the chemical shifts of the alpha and amide protons of the muramyl pentapeptides used was extremely helpful. In addition, the model we used revealed two more, previously not described, hydrogen bonds formed with vancomycin by the amino group of L-lysine from muramyl pentapeptide and an additional proton–proton through-space interaction (NOESY spectrum) affecting vancomycin-muramyl pentapeptide complex formation. We confirmed this model in in silico modeling using molecular dynamics and trajectory analysis.

## Data Availability

Not applicable.
